# A Self-Reference Interference Sensor Based on Coherence Multiplexing

**DOI:** 10.3389/fchem.2022.880081

**Published:** 2022-03-23

**Authors:** Ying Shen, Zeyu Huang, Feng Huang, Yonghong He, Ziling Ye, Hongjian Zhang, Cuixia Guo

**Affiliations:** ^1^ School of Mechanical Engineering and Automation, Fuzhou University, Fuzhou, China; ^2^ Shenzhen Key Laboratory for Minimal Invasive Medical Technologies, Institute of Optical Imaging and Sensing, Tsinghua Shenzhen International Graduate School, Tsinghua University, Shenzhen, China

**Keywords:** phase-sensitive interferometry, biosensing, differential measurement, biomolecular interaction, label-free detection

## Abstract

Interferometry has been widely used in biosensing due to its ability to acquire molecular affinity and kinetics in real-time. However, interferometric-based sensors are susceptible to environmental disturbances, including temperature and non-specific binding of target molecules, which reduces their detection robustness. To address this shortcoming, this paper proposes a self-referencing interference sensor based on coherence multiplexing to resist environmental disturbances. The proposed sensor can address temperature and non-specific binding, but it is not limited only to these types of disturbances. In the proposed sensor design, each sensor signal is encoded using a specific optical path difference determined by the optical thickness of a sensor chip. In addition, two sensor signals for disturbances tracking and biomolecule detection are detected simultaneously without additional cost to the second spectrometer and then differenced to achieve real-time self-reference. The temperature fluctuations experiments and specific binding experiments of protein A to IgG are performed to verify the performance of the proposed sensor. The results demonstrate that the proposed sensor can eliminate non-specific binding and temperature disturbances in real-time during biomolecule detection, achieving higher detection robustness. The proposed sensor is suitable for applications that require large-scale testing of biomolecular interactions, such as drug screening.

## Introduction

The development of simple, sensitive, and rapid molecular detection methods is of great importance to many fields, including medical evaluation, drug screening, and environmental applications. Molecular detection methods can provide accurate and fast drug sensitivity results, providing new tools for better understanding of drug-resistant tuberculosis (Rubin, 2018), and can also identify bacteria (Tardif et al., 2016) and detect specific micro-molecules in areas of water contamination (Dandapat et al., 2016). Hence, the molecular detection and identification methods have high application importance.

The molecular detection and identification methods can be roughly categorized into labeled methods and label-free methods. Due to high sensitivity, labeled methods, e.g., fluorescence (Sun et al., 2018; Burg et al., 2019), chemiluminescence (Jin et al., 2017; Li et al., 2017), enzyme-linked immunosorbent assay (ELISA) (Engvall and Perlmann, 1971), have been used in many sensing measurements. However, due to the complex structure and reactivity of proteins, an approach of adding additional reagents to improve the accessibility of observation could cause changes in the properties of a target molecule (MacBeath, 2002). In addition, for labeled methods, it is difficult to provide real-time insight into the molecular binding process and allow visualization of the molecular binding kinetics. Due to these disadvantages of the labeled methods, an increasing number of label-free methods have been used in biosensing applications, including optical waveguide lightmode spectroscopy (OWLS) (Orgovan et al., 2014), surface plasmon resonance (SPR) (He et al., 2016; Liu et al., 2017), ellipsometry (Demircioglu et al., 2017), and biolayer interferometry (BLI) (Sztain et al., 2021). However, these methods not only require customized and expensive substrates but also can difficultly achieve *in-situ* detection. For instance, SPR substrates need to be coated with an expensive gold film (Hobbs et al., 2016), whose thickness has to be precisely controlled at the nm level.

Recently, phase-sensitive interferometry (Joo et al., 2009; Chirvi et al., 2012; Ryu et al., 2014; Merryweather et al., 2021) has attracted great attention as a phase image technique. Spectral-domain phase-sensitive interferometry (SD-PSI) has been used as a quantitative phase imaging method in biosensing applications. The SD-PSI can acquire the molecular layer thickness changes caused by binding the target molecules to the probe surface in real-time. A fiber optic molecular sensor based on the SD-PSI can be used to monitor biomolecules *in situ* (Guo et al., 2018). Unlike other label-free detection methods, the SD-PSI does not require custom and expensive sensor substrates, such as gold-plated trigonal prisms, which are used in the SPR (Wu et al., 2010). The previous studies (Joo et al., 2009; Chirvi et al., 2012; Ryu et al., 2014) have demonstrated that laboratory-grade or off-the-shelf glass of a suitable thickness can be used as a sensor chip, and such a design is low-cost and simple to prepare. However, this technique is susceptible to disturbances induced by the temperature response of a sensor chip, non-specific binding of target molecules, and sample background. The previous experiments have required keeping the sensor and buffer solution at a constant temperature as much as possible (Chirvi et al., 2012), which increased experimental complexity and reduced experimental robustness.

The previous solutions are easily affected by different disturbances in biosensing, including temperature response and non-specific binding of target molecules. To overcome this shortcoming, this paper proposes a self-reference interference sensor based on coherence multiplexing, which can provide a differential measurement result with the phase change caused by binding of target molecules. The proposed sensor establishes referential and measuring paths with different optical path differences (OPDs) for disturbance tracking and biomolecule detection, thus realizing a self-reference measurement. The advantage of coherence multiplexing is that it allows simultaneous detection of two OPD-coded sensing signals without adding an expensive additional detection element. In addition, compared to the time-coded sequential measurement self-reference methods, the coherence multiplexing coding methods allow simultaneous biomolecule detection and disturbance dynamics tracking in both paths, thus improving detection robustness. The proposed sensor can be used as a label-free sensor with the advantages of weak temperature sensitivity, low non-specific binding, and picometer-level thickness sensitivity. In addition, the proposed sensor requires using only an ordinary optical glass as a detection substrate, which is low-cost and simple to manufacture.

## Principle and Proposed Sensor Design

**FIGURE 1:**
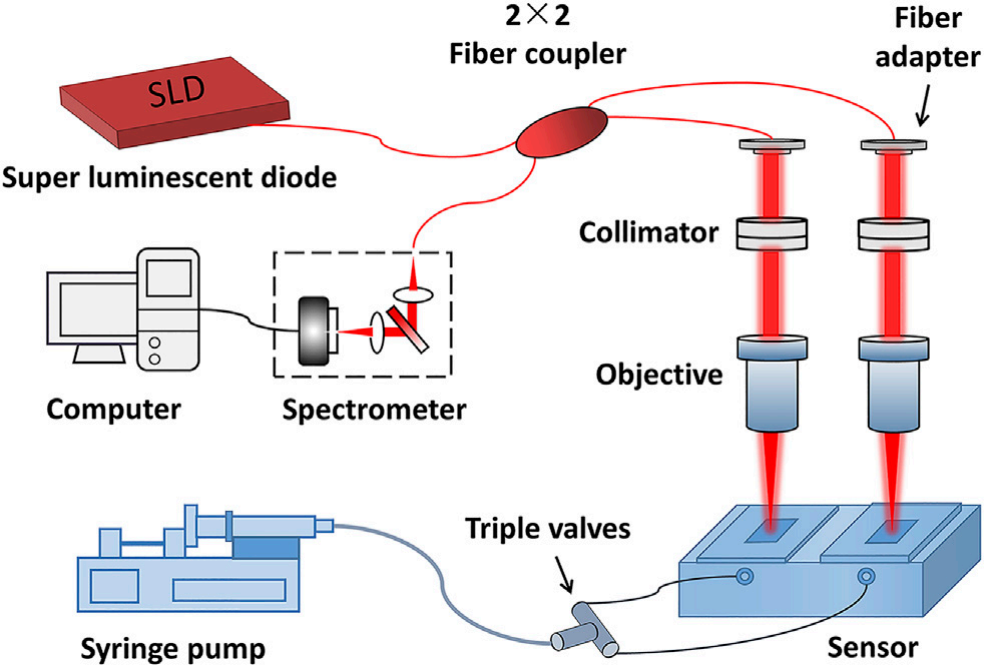
The self-reference setup of biomolecule detection.

The self-reference setup of the biomolecule detection method proposed in this study is presented in Figure 1. The setup is based on the SD-PSI. A super-luminescent diode provides the incident light with a central wavelength of 1,310 nm and a bandwidth of 75 nm. The two detection paths are prepared by a single-mode fiber-based 2 × 2 coupler and two sensor chips with different thicknesses. Each path can be considered as a low-coherence interferometer with a common path, where the reference light is reflected from the upper surface of a sensing chip, and the sample light is reflected from the contact surface between the sensor chip and solution. The interference signal of each path is encoded with a specific OPD, which is equal to the optical thickness of the corresponding sensor chip, i.e., the product of refractive index and thickness. The two signals were recorded by a homemade transmission grating structure-based spectrometer with a spectral resolution of 0.07 nm and a spectral measurement range of 1,240–1,380 nm, which was then decomposed from the superimposed interferometric spectra according to the difference in OPD values. The OPD between the two paths needs to be greater than the measuring range of the SD-PSI so that to avoid interference between the two paths’ beams. Sample channels are constructed by using epoxy glue to combine two different initial thicknesses of sensing chips (0.17-mm and 0.2-mm glass flakes) with a fluid chip (built-in flow channel and reaction chamber).

**FIGURE 2:**
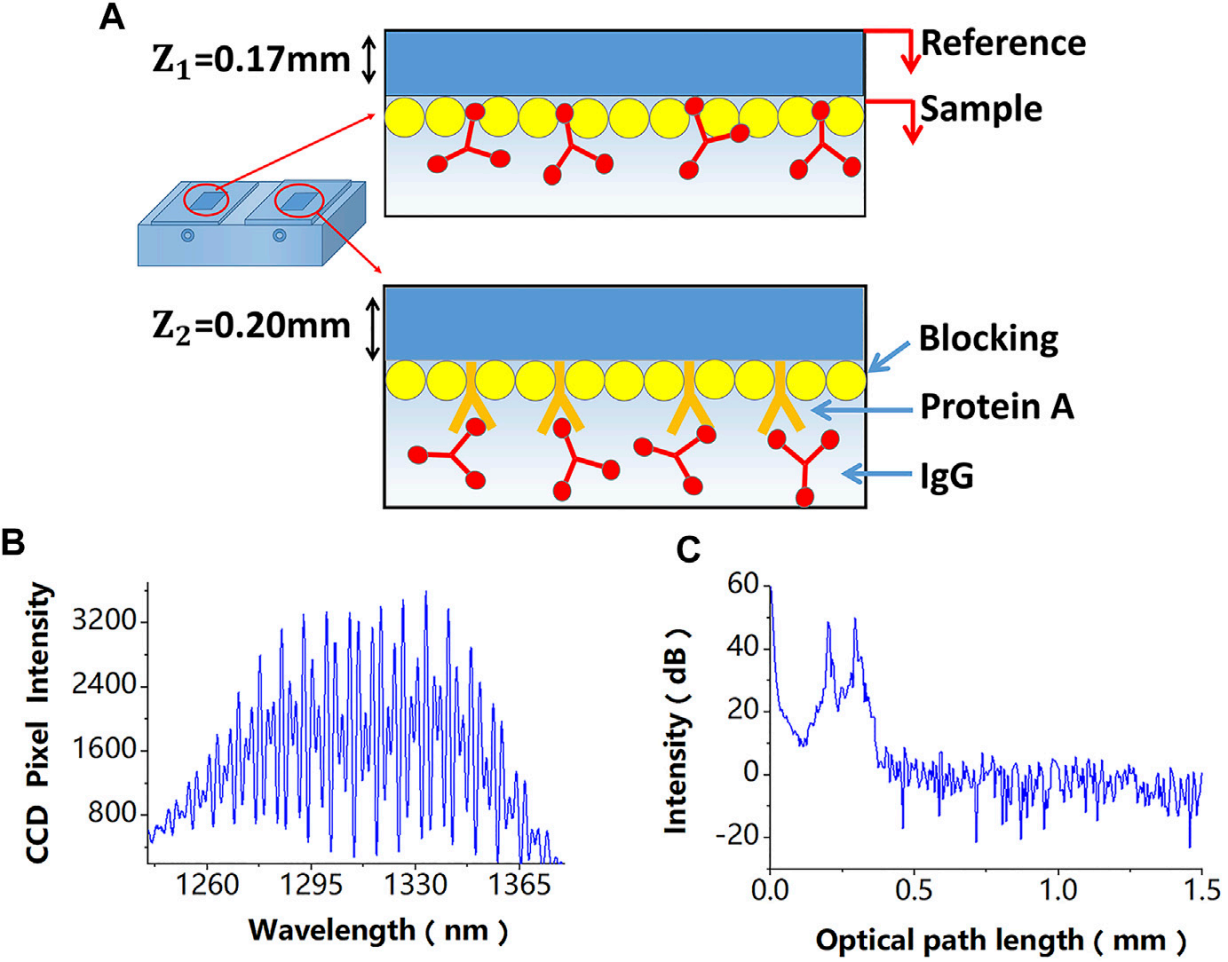
**(A)** A differential-path modification method of sensing chips, the difference of these modification processes is that the referential path does not modify the probe molecule. **(B)** Superimposed interferometric spectra obtained by the SD-PSI. **(C)** Signal peaks corresponding to different OPDs obtained after performing the fast Fourier transform algorithm on the two superimposed interferometric spectra.

The binding of a molecule to be detected to the probe molecule on the sensing chip surface changes the sensing chip thickness, which causes a phase shift of the interference signal. Disturbances of a sensing chip, including non-specific binding and temperature response, can also induce changes in the interference signal phase. The two detection paths in the self-reference system, denoted as path 1 (P1) and path 2 (P2), are used to detect phase variations induced by the biomolecule detection and disturbances during the biomolecule detection process, respectively. Finally, the self-reference molecule detection is achieved by performing the difference operation between the phases of the two OPD-encoded signals. The basic principle of the self-reference interference sensor based on coherence multiplexing is shown in Figure 2.

In the proposed sensor design, a differential-path modification method for sensing chips is shown in Figure 2A, which is described in detail in the next section, is used. The two superimposed interferometric spectra signals detected by a spectrometer are shown in Figure 2B, which can be expressed as follows:

$$I\left(k\right)=2\beta S\left(k\right)\sqrt{R_{r}R_{s}}\cos\left(2k\left(Z_{10}+Z_{20}\right)+\phi_{1}^{\prime }\left(t\right)+\phi_{2}^{\prime }\left(t\right)\right)$$ (1)

where *k* is the wavenumber, *β* is the beam splitting ratio of the fiber coupler, and *S*(*k*) is the spectral density of the Gaussian-type light source; $R_{r}$ and $R_{s}$ are the reflectance values of the upper surface of the sensing chip and the sample layer, respectively; $Z_{10}$ and $Z_{20}$ are the initial optical thicknesses of the sensing chips, corresponding to the encoded signals; $\phi_{1}^{\prime }\left(t\right)$ is the phase induced by the biomolecule detection in molecular detection path P1 due to the true specific molecular binding, as well as temperature and non-specific disturbances; $\phi_{2}^{\prime }\left(t\right)$ is the phase induced by temperature and non-specific disturbances in disturbances tracking path P2.

Since paths P1 and P2 have different OPDs, their signal peaks can be obtained, as shown in Figure 2C, after performing the fast Fourier transform algorithm on the two superimposed interferometric spectra. The phases of the space-domain interference signals at these two peaks, which also include phase variations induced by biomolecule detection and disturbances, can be calculated by:

$$\phi_{c=1,2}\left(t\right)=\tan^{-1}\left\{\frac{Im\left(I_{c=1,2}\left(Z\right)\right)}{Re\left(I_{c=1,2}\left(Z\right)\right)}\right\}=\phi_{c=1,2T}\left(t\right)+\phi_{Bc=1,2}\left(t\right)$$ (2)

where $\phi_{c=1,2T}\left(t\right)$ is the phase change induced by the temperature response of the sensor chips in paths P1 and P2, and it is in line with the initial optical thickness of the sensing chips and temperature and can be approximately expressed as $\phi_{c=1,2T}\left(t\right)\approx TZ_{c=1,20}$ .

Thus, the temperature response of the molecular detection path P1 can be calculated by linearly fitting the temperature response of the disturbances tracking path P2, which can be expressed as follows:

$$\phi_{1T}\left(t\right)=a\phi_{2T}\left(t\right)+b$$ (3)

where a denotes the temperature correction factor, and b is the temperature compensation factor.

Different modification processes are used in different thickness chips of the proposed sensor; the difference of these processes is that the referential path does not modify the probe molecule. The proposed design allows tracking the total phase change $\phi_{B1}\left(t\right)$ due to specific binding and non-specific disturbance in P1, while the non-specific disturbance $\phi_{B2}\left(t\right)$ is simultaneously tracked in P2. Then, $\phi_{Bc=1,2}\left(t\right)$ is differenced in real-time to obtain the corrected specific binding by:

$$Z_{corrected}=\frac{\phi_{B1}\left(t\right)-\phi_{B2}\left(t\right)}{2k_{0}}=\;\frac{\phi_{1}\left(t\right)-\phi_{2}\left(t\right)-\left(a-1\right)\phi_{2T}\left(t\right)-b}{2k_{0}}$$ (4)

where $k_{0}$ is the central wavenumber of the light source, and it is given by $k_{0}=2\pi /\lambda_{0}$ , where $\lambda_{0}$ is the central wavelength of the broadband light source.

In the experiment, $\phi_{c=1,2T}\left(t\right)$ denotes the average phase change calculated from the stable data before performing molecular detection; $\phi_{c=1,2T}\left(t\right)$ is used in Eq. 3 to obtain a and b .

## Results and Discussion

### Weak Temperature Sensitivity

It is well known that using glass as a sensing chip has a low cost and is simple to prepare. The process of glass surface modification has been established and widely used in the preparation of molecular-level sensors (Joo et al., 2009). However, such sensing chips are sensitive to the ambient temperature. Depending on the composition, the thermal expansion coefficient is roughly equal to $\left[\left(5.8\;∼\;150\right)\times 10^{-7}\right]$ per Kelvin temperature (K). For instance, a 0.20-mm laboratory-grade glass sheet used in this study causes a thickness change of about 1 nm/K, which is unfavorable for sub-nanometer thickness measurements.

**FIGURE 3:**
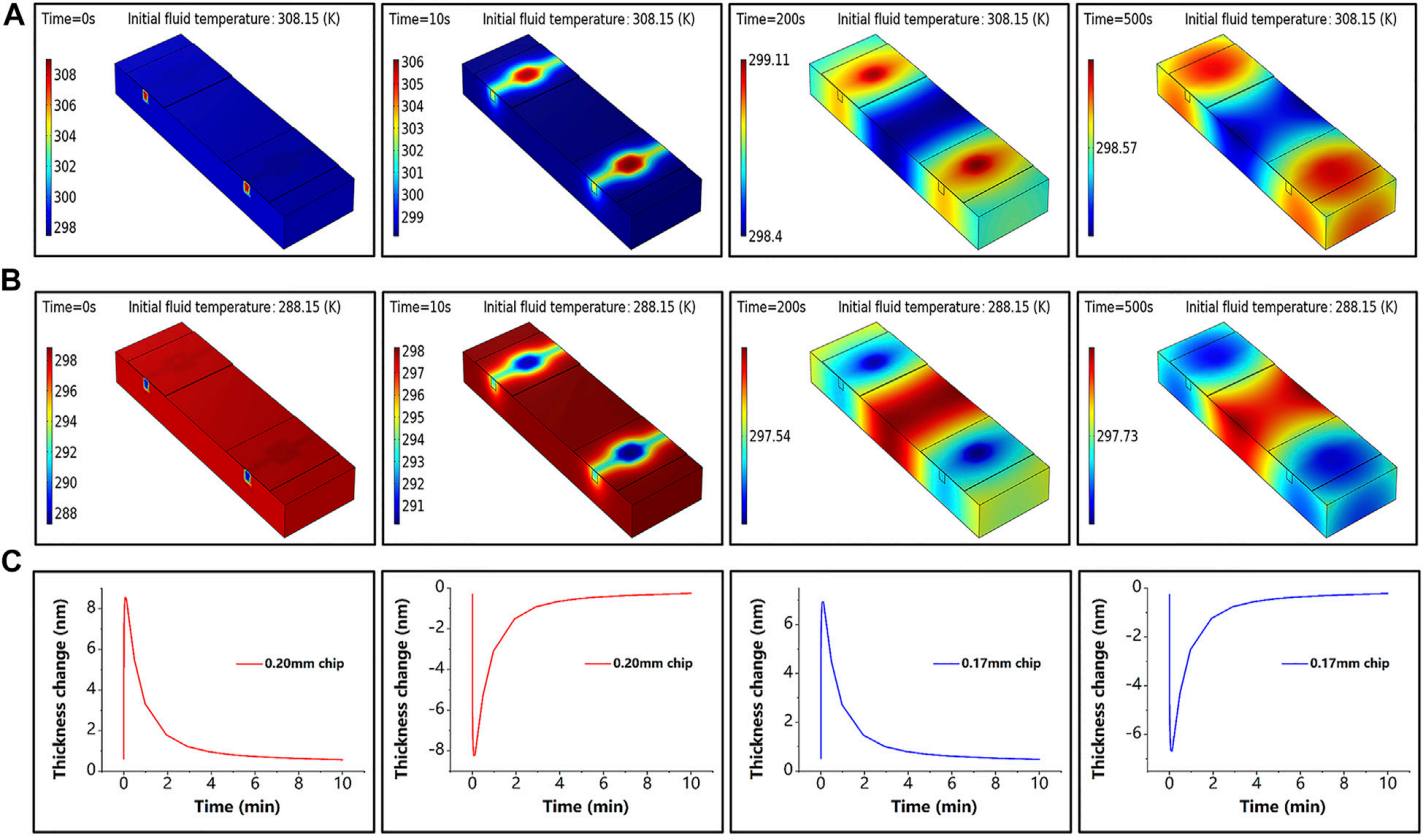
Simulation results. **(A)** Sensor temperature at different moments under the initial temperature of fluid of 308.15 K when the simulated ambient temperature increases. **(B)** Sensor temperature at different moments under the initial temperature of fluid of 288.15 K when the simulated ambient temperature decreases. **(C)** Thickness variation at the center point of the sensing chip for different initial thicknesses during simulated temperature rise and fall; the red line corresponds to a thickness of 0.20 mm and the blue line to a thickness of 0.17 mm.

To confirm that the sensing chip made of glass is sensitive to temperature, the heat transfer process was simulated by COMSOL software. The effect of the ambient temperature change of room temperature (298 ± 10 K) on the glass chip was simulated by changing the fluid’s initial temperature. The simulation results are presented in Figure 3, where Figures 3A,B show the sensor temperature distribution at different moments during the temperature rise and fall, and Figure 3C shows the thickness variation of the sensing chip with different initial thicknesses, which is generated by the increase or decrease of the ambient temperature. In the simulation experiment, we measure the temperature variation ΔT at the center point of the sensing chip and set the thermal expansion coefficient δ as $\left(50\times 10^{-7}\right)\;/K$ . The thickness variation curve is obtained based on $\Delta Z=\Delta T\times \delta \times Z_{0}$ . Although the thickness converges quickly to the initial value again, it is a challenge to keep the thickness constant. Since this thickness variation is related to temperature variation, reducing the temperature sensitivity in sensing measurements at high accuracy levels is a problem that needs to be solved.

**FIGURE 4:**
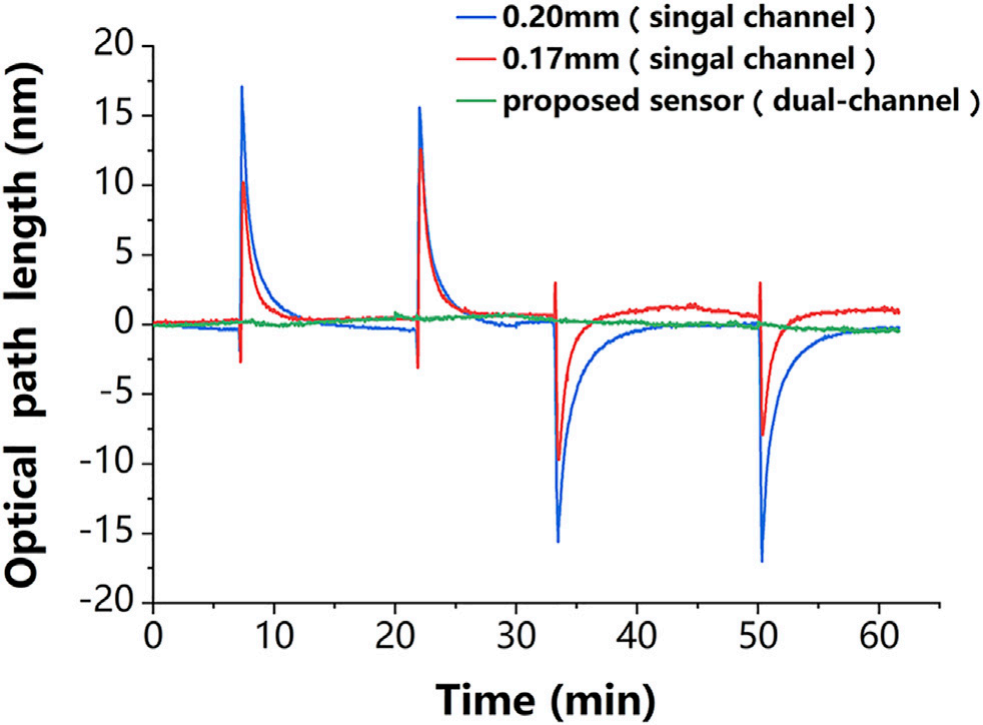
Experimental results. The thickness variation of the chips in the single-channel sensor (red and blue lines) is very consistent with temperature variation in Figure 3C, which proves that this sensing chip is sensitive to the temperature disturbance. The phase change of the proposed dual-channel sensor (green line) is about one-tenth of that of the single-channel sensor, which proves that it is less sensitive to temperature disturbance.

In addition, the robustness of the proposed sensor against temperature fluctuations was experimentally evaluated. The experiments followed the control principle and took full advantage of a dual-channel sensor. For a clearer demonstration, the temperature change in the experiment was deliberately magnified. The change in ambient temperature was simulated by simultaneously and repeatedly passing the deionized water at the ambient temperature that was 10 K above (or below) room temperature into the reaction chamber. The thickness variation curves of the sensing chip of the single-chamber sensor and the proposed dual-chamber sensor are presented in Figure 4, where it can be seen that compared to the proposed sensor, the sensing chip of the conventional sensor was more sensitive to temperature changes, and its thickness change curve was very consistent with that in Figure 3C, indicating that the glass chip was sensitive to temperature; the thickness change curves of the two chips were different due to the difference in their initial thicknesses. It is worth mentioning that the results of the proposed sensor were obtained by linearly fitting the results of single-chambers, which was an improvement of the traditional method. The standard deviation of the phase changes obtained from the experimental results was used to evaluate the temperature sensitivity of the self-reference-type sensor. The experimental results showed that the average value of the conventional sensor was about $2.5\times 10^{-2}\;\mathrm{rad}$ , which corresponded to the optical thickness of 2.61 nm, and that of the proposed sensor was $2.9\times 10^{-3}\;\mathrm{rad}$ , which corresponded to the optical thickness of 0.30 nm. The results demonstrated good robustness of the proposed sensor to temperature changes.

### Track and Suppress Non-Specific Binding

The non-specific binding affects detection results of molecular measurements, and conventional blocking methods cannot completely eliminate non-specific binding. However, the proposed sensor monitors the non-specific binding process using a differential-path detection method and has the ability to correct for specific binding.

To verify the non-specific binding suppression performance of the proposed sensor, first, the stability of phase characterization was examined by injecting only a phosphate-buffered saline (PBS) buffer for 20 min at room temperature. The total phase variation of $4\times 10^{-4}\;\mathrm{rad}$ in the first 20 min, which corresponded to the optical thickness variation of 41 p.m., indicated that the phase characterization capability of the proposed sensor was stable enough. Also, the standard deviation of a phase variation of $6.7\times 10^{-5}\;\mathrm{rad}$ , which corresponded to the optical thickness variation of 6.9 p.m., indicated the pico-meter-level thickness sensitivity of the proposed sensor.

Next, one of the glass chips with a 0.20-mm thickness was modified. First, a dopamine-Tris solution of 2 mg/ml was used to form a thin adhesion layer by self-polymerization on the binding surface to improve the biocompatibility of the binding surface. Then, the dopamine layer was non-specifically modified with the protein A (0.5 mg/ml), which acted as probe molecules to capture the target analyte. Finally, the non-specific binding site on the dopamine layer was blocked by passing through the protein-free blocking solution. To reduce the non-specific binding of the target analyte to the dopamine layer, smaller blocking molecules filled the dopamine layer between the protein A molecules. The PBS was introduced to the fluid chip to flush the unbound molecules before passing through a new solution. In addition, an almost identical method was used to modify another glass chip; the only differences were that no protein A solution was passed through, and the dopamine layer directly adhered with the protein-free blocking solution.

**FIGURE 5:**
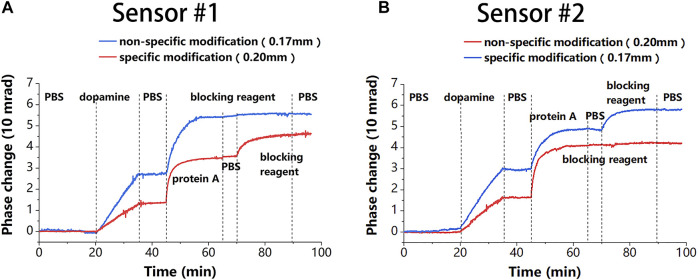
The modification results of the two sensors, where the blue line corresponds to the 0.17-mm chip and the red line corresponds to the 0.20-mm chip. **(A)** The modification result of sensor #1, where a 0.20-mm chip is used for specific binding and a 0.17-mm chip for non-specific binding. **(B)** The modification result of sensor #2, where a 0.17-mm chip is used for specific binding and a 0.20-mm chip for non-specific binding.

The modification processes of the two sensing chips were then exchanged to obtain the second proposed sensor. Due to the modification processes were exchanged, the probe molecules were present on different thickness chips in the two sensors. This difference between the two sensors was used to verify channel uniformity of the proposed sensor, which is a prerequisite for achieving undifferentiated sensor detection. As shown in Figures 5A,B, the phase changes of the two sensors under the same reaction were almost identical during specific modifications monitored by different sensing chips (0.17 mm for the blue and 0.20 mm for the red), except for the growth of dopamine. The same results were achieved for non-specific modifications. This shows that the chip thickness does not affect the molecular reaction, thus proving the channel uniformity of the proposed sensor. The results also demonstrated that the total increase in phase caused by blocking molecules under the specific modification was less than that under the non-specific modification. This could be because protein A preoccupied most of the binding sites.

**FIGURE 6:**
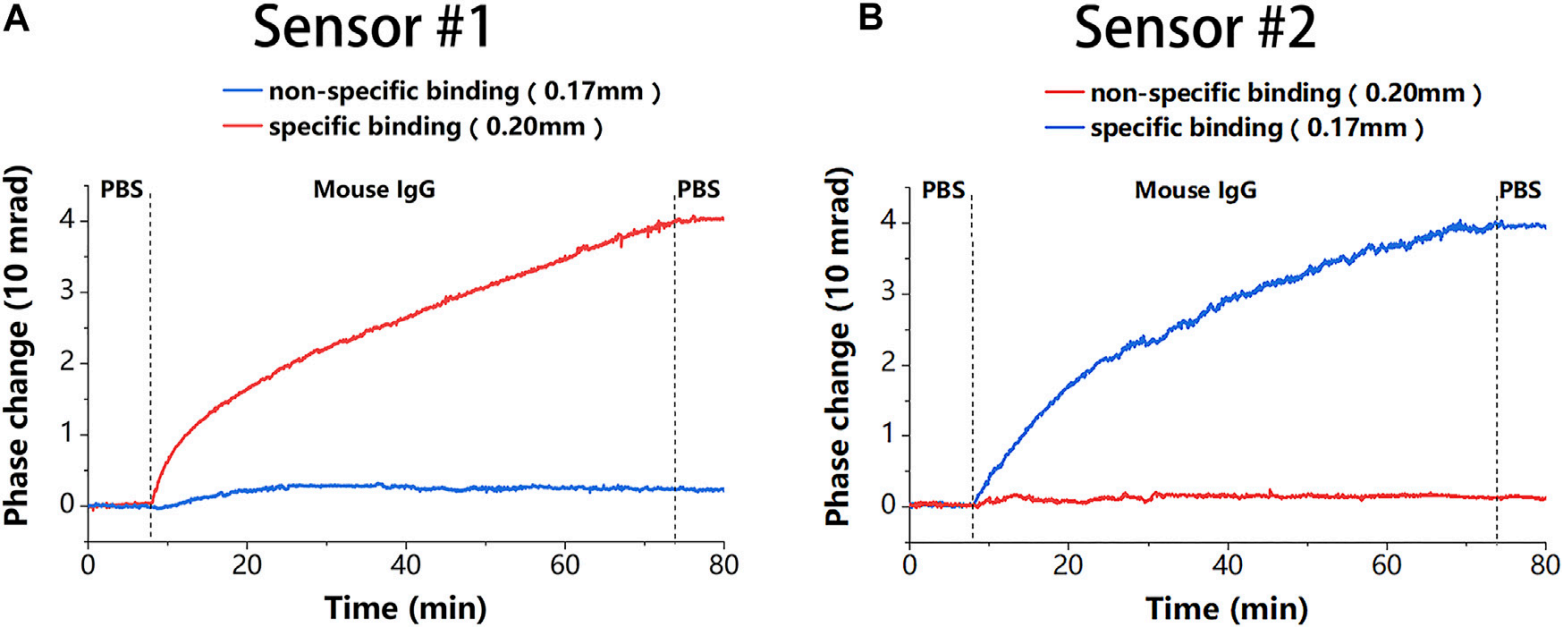
The binding results of the two sensors. **(A)** Binding results corresponding to Figure 5A in sensor #1. **(B)** Binding results corresponding to Figure 5B in sensor #2.

After modification, 20 μg/ml of mouse IgG solution was used to demonstrate the detection capability of the proposed sensor. To reduce the effect of phase noise further, the average phase of 1,000 neighboring measurement points was calculated and used as a phase at that moment in the experiment, and the response time of the system was 27.8 ms, which satisfied the real-time requirement. The results in Figures 6A,B show that the binding detected under different initial thicknesses of the sensing chip was almost the same, which further demonstrates good channel uniformity of the proposed sensor. This uniformity makes the proposed sensor more flexible and easier to prepare. The phase change of about $4\times 10^{-2}\;\mathrm{rad}$ caused by specific binding was mainly due to the binding of the mouse IgG to protein A. The non-specific binding caused a phase change of about $2\times 10^{-3}\;\mathrm{rad}$ , which could be because the dopamine layer was not completely closed by the blocking reagent molecules, thus allowing IgG to adhere to the dopamine layer. This change was independent of the probe.

**FIGURE 7:**
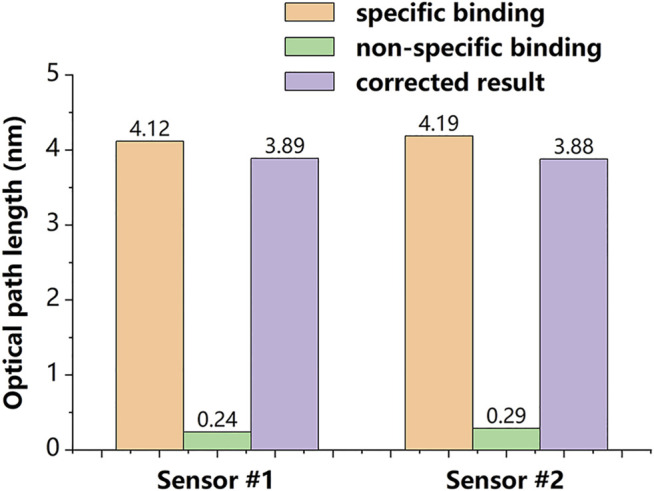
Relationship between the amount of binding and correction.

We calculated the average value of the peak phase Δϕ in Figure 6 for each reaction after the reaction was completed, and subsequently calculated the growth of optical path length due to specific binding and non-specific binding by $\Delta Z=\Delta \phi /2k_{0}n$ . To eliminate this variation, the stable data were used in the first 5 min to obtain the correction factor a and compensation factor *b*, which are given by Eq. 3 and used in Eq. 4 to correct specific binding. The relationship between the binding and the corrected result is displayed in Figure 7. The results demonstrated the proposed sensor has the capability to track and suppress non-specific binding, and this capability offers the potential for more accurate drug screening.

**FIGURE 8:**
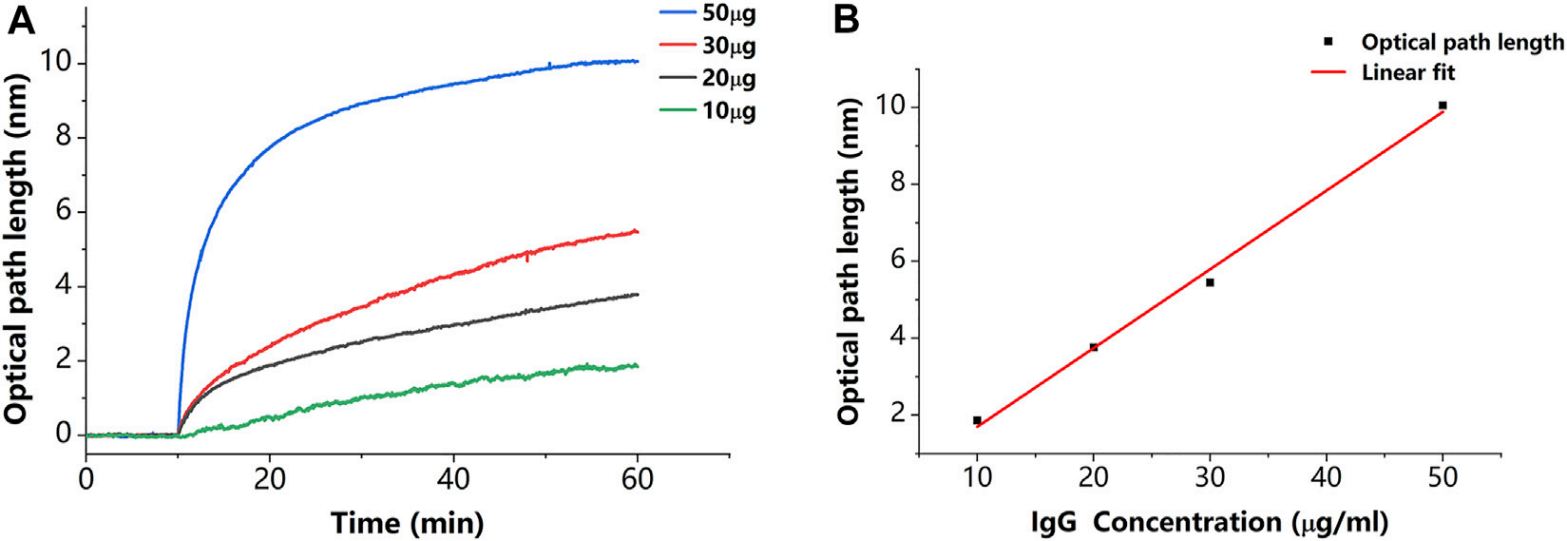
**(A)** Results of binding of IgG to protein A in different concentrations. **(B)** Relationship between thickness variation and IgG concentration. Each plotted data point in Figure 8B is averaged from three measurements.

In addition to verifying that the sensor has the described capabilities, we estimated the sensitivity of the described sensor. As shown in Figure 8A, we set a concentration gradient to verify that the specific binding of IgG to protein A causes a proportional variation in thickness. The thickness variations caused by 10, 20, 30, and 50 μg/ml concentrations of IgG molecules are 1.86, 3.76, 5.45, and 10.06 nm, respectively. Each plotted data point in Figure 8B is averaged from three measurements. The results of the linear fit are shown in Figure 8B with a slope of 0.205 nm/(μg/ml). This slope can be used to estimate the minimum detectable concentration, which indicates the sensitivity of the described sensor. The minimum detectable concentration is calculated by $D_{lim}=3\sigma_{s}/\left(\delta h/\delta c\right)$ , where δh/δc is the slope of linear fit and $\sigma_{s}$ is the standard deviation of the thickness after binding is completed. The standard deviation of the thickness from the data of 50 μg/ml is calculated as 0.011 μg/ml. Therefore, the sensitivity of the sensor is calculated as 0.16 μg/ml.

## Conclusion

This paper presents a sensor based on coherence multiplexing, which can be used to resist different disturbances in biosensing applications, including temperature and non-specific binding disturbances. The proposed sensor has two reaction chambers and sensing chips of different thicknesses, which define two interferometric paths with different OPDs. This allows the proposed sensor to achieve self-reference based on coherent multiplexing. The proposed sensor is suitable for applications that require large-scale testing of biomolecular interactions, such as drug screening. Using fiber optic-based glass as a sensor chip reduces the cost of the proposed sensor. This technology can also be used in other sensing applications, such as multi-channel pressure sensing systems.

## Data Availability

The raw data supporting the conclusion of this article will be made available by the authors, without undue reservation.
