# Corrigendum: A self-reference interference sensor based on coherence multiplexing

**DOI:** 10.3389/fchem.2024.1515546

**Published:** 2024-11-06

**Authors:** Ying Shen, Zeyu Huang, Feng Huang, Yonghong He, Ziling Ye, Hongjian Zhang, Cuixia Guo

**Affiliations:** ^1^ School of Mechanical Engineering and Automation, Fuzhou University, Fuzhou, China; ^2^ Shenzhen Key Laboratory for Minimal Invasive Medical Technologies, Institute of Optical Imaging and Sensing, Tsinghua Shenzhen International Graduate School, Tsinghua University, Shenzhen, China

**Keywords:** phase-sensitive interferometry, biosensing, differential measurement, biomolecular interaction, label-free detection

In the published article, there was an error in the **Funding** statement. The grant number for the National Natural Science Foundation of China was incorrect. The correct statement appears below:

“This work was financially supported by the National Natural Science Foundation of China (62105068), Ministry of Education Science and Technology Industry-University Cooperative Education Program (202102153072), Educational Research Project for Young and Middle-aged Teachers of Fujian Provincial Education Department (JAT200009), and Fuzhou University Research Start-up funding (GXRC-21019).”

The authors apologize for this error and state that this does not change the scientific conclusions of the article in any way. The original article has been updated.

